# Refractory Heparin-Induced Thrombocytopenia With Cerebral Venous Sinus Thrombosis Treated With IVIg, Steroids, and a Combination of Anticoagulants: A Case Report

**DOI:** 10.1177/2324709619832324

**Published:** 2019-04-02

**Authors:** Mia Gonzales, Amrish Pipalia, Andrew Weil

**Affiliations:** 1 East Carolina University, Greenville, NC, USA; 2 Vidant Medical Center, Greenville, NC, USA

**Keywords:** heparin-induced thrombocytopenia, cerebral venous sinus thrombosis, IVIg, refractory HIT, autoimmune HIT

## Abstract

Heparin-induced thrombocytopenia (HIT) type II is caused by antibody production that bind complexes between heparin and platelet factor 4 leading to platelet consumption and thrombosis. In a small subset of cases referred to as autoimmune HIT, the antibodies activate platelets even in the absence of heparin. Refractory HIT is a type of autoimmune HIT in which thrombocytopenia persists for weeks after heparin discontinuation and carries increased risk for thrombosis and more severe thrombocytopenia. We present a case of refractory HIT with cerebral venous sinus thrombosis (CVST) that was successfully treated with a change in anticoagulant alongside steroids and a second trial of intravenous immunoglobulin (IVIg).

## Introduction

Heparin-induced thrombocytopenia (HIT) type II is caused by immunoglobulin G antibodies binding to complexes formed between heparin and platelet factor 4, which triggers platelet activation and clearance.^1^ Platelet activation can lead to thrombosis, which carries a mortality rate of 8% to 20% regardless of therapy.^2,3^ In a subset of cases referred to as autoimmune HIT, antibodies activate platelets even in the absence of heparin.^4^ Refractory HIT is a type of autoimmune HIT in which thrombocytopenia persists after heparin discontinuation. In this article, we present a case of refractory HIT with cerebral venous sinus thrombosis, which was successfully treated with a combination of direct thrombin inhibitors (DTIs), steroids, and intravenous immunoglobulin (IVIg).

## Case Presentation

A 46-year-old woman underwent simple mastectomy for treatment of breast cancer at an outside hospital. Her admission platelet count was 335 000/µL, and her postoperative course was uncomplicated. She was discharged on low-molecular-weight heparin (LMWH) for venous thromboembolism (VTE) prophylaxis on postoperative day 2. After 8 days of LMWH therapy, she presented to the emergency department with a progressively worsening headache. She had associated blurry vision but no other neurological deficits. Platelet count at this presentation was 12 000/µL. Computed tomography venography of the head revealed thrombosis extending from the superior sagittal sinus into the right sigmoid sinus. The 4T score^5^ was calculated as 7 and HIT was later confirmed with a positive heparin-induced platelet antibody ELISA (enzyme-linked immunosorbent assay) screen (2.69 OD) and serotonin release assay (100% at 0.1 IU/mL and 99% at 0.5 IU/mL).

**Figure 1.**
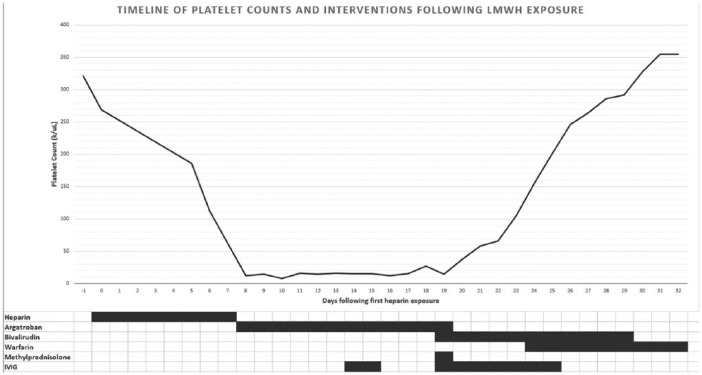
Platelet count during hospital course.

All heparin products were discontinued and argatroban was initiated. Although a therapeutic partial thromboplastin time (PTT) was maintained for 7 days, there was slight extension of thrombosis and no improvement in platelet count, which suggested a refractory variant of HIT. IVIg was administered for 2 days at 0.7 g/kg/day with minimal improvement of platelet count. Platelet counts continued to remain low at 14 days following LMWH discontinuation. At this time, argatroban was switched to bivalirudin, methylprednisolone 1000 mg was administered once, and IVIg was reinitiated at 0.4 g/kg/day for 7 days. Her platelet counts subsequently demonstrated a steady rise, reaching normal levels within 5 days (Figure 1). She was transitioned to warfarin. On discharge, her platelet count was 355 000/µL.

## Discussion

### Autoimmune and Refractory HIT

Autoimmune refractory HIT occurs rarely, although the incidence is unknown. It can manifest as refractory HIT, delayed-onset HIT, spontaneous HIT, or other rare clinical entities.^4^ These syndromes occur when autoantibodies are able to bind platelet factor 4 and activate platelets independently of heparin.^4^ In refractory HIT, thrombocytopenia persists or worsens for >1 week after discontinuing heparin, and there is increased risk for thrombosis.^1,4,6,7^ In addition, Doucette et al indicate that severe thrombocytopenia (<20 000/µL), which is not typical of HIT, occurs in more than half of cases with refractory HIT.^7^ Our patient presented with severe thrombocytopenia that persisted for 10 days despite standard therapy. She also developed extension of her thrombosis during this period.

### Risk Assessment, Monitoring, and Prevention

In a recently updated set of guidelines published by the American Society of Hematology (ASH), patients who have an intermediate (0.1% to 1.0%) or high (>1.0%) risk of developing HIT should undergo platelet count monitoring.^8^ Intermediate-risk populations include medical and obstetric patients receiving unfractionated heparin and patients receiving LMWH after major surgery or major trauma.^8^ This patient is considered to have intermediate risk of developing HIT due to malignancy and for recently undergoing mastectomy. She was placed on VTE prophylaxis with LMWH after mastectomy at an outside hospital, but platelet counts were not monitored every 2 to 3 days as recommended by the ASH.^8^ The benefit of monitoring intermediate-risk patients is early detection of thrombocytopenia, but the ASH states the certainty in the effects of platelet monitoring is very low.^8^

Prevention of HIT is only possible through reduced exposure to heparin. Growing research has demonstrated noninferiority and cost savings, and in some cases superiority, of direct oral anticoagulants (DOACs) compared with LMWH in the prevention of VTE in both medical and postoperative hospitalized patients.^9-12^ For patients with intermediate to high risk of HIT, such as our patient, it may therefore be advantageous to prefer a DOAC over unfractionated heparin or LMWH for VTE prophylaxis.

### Choice of Anticoagulant

The standard anticoagulants used in HIT are the DTIs, argatroban, and bivalirudin. These are commonly used due to short half-lives, readily available albeit indirect monitoring with PTT, and familiarity among providers. Our patient was initiated on argatroban, which has been shown by Skrupky et al to achieve similar therapeutic anticoagulation, clinical outcomes, and safety as bivalirudin.^13^ Nevertheless, it carries the potential for argatroban-induced thrombocytopenia^14^ and an interference with international normalized ratio, which demands close clinical monitoring and complicates the transition to an oral vitamin K antagonist.^15^ A study by Bain et al, however, contradicts findings by Skrupky et al and suggests that bivalirudin may have lower bleeding rates and a quicker time to therapeutic PTT compared with argatroban.^13,15^ Our patient was switched from argatroban to bivalirudin after her platelet count did not respond to argatroban.

Other anticoagulants used in the treatment of HIT include the indirect factor Xa inhibitors danaparoid and fondaparinux and the DOACs. Both danaparoid and fondaparinux are often preferred due to their reliable direct monitoring with anti–factor Xa assays and much longer half-lives than the DTIs, which reduces rebound hypercoagulability. Both are also available in subcutaneous form, but fondaparinux has no parenteral option. They suffer from a lack of reversal options, renal-dependent dosing, and a debated risk with danaparoid of HIT exacerbation due to cross-reactivity to HIT antibodies.^16,17^ Additionally, danaparoid is not available in the United States. DOACs such as apixaban, rivaroxaban, and dabigatran are emerging alternative therapies that are promising for their ease of use and shortened hospital stays. There are very few studies, most of which are retrospective and confounded by initial parenteral treatment, but the data show comparable safety and efficacy.^17-20^ However, DOACs suffer from limited availability and high cost of reversal agents.^21,22^

### Intravenous Immunoglobulin

The premise for the use of IVIg to treat HIT stems from its use in multiple other immune-mediated diseases including some thrombocytopenia syndromes.^23^ There are <30 case reports of IVIg used to successfully treat refractory HIT.^7,23,24^ Of these cases, most had a rapid response in platelet count 1 to 5 days after administration of 1 to 2 doses of IVIg.^7,24^ Park et al performed in vitro studies with the serum of patients with refractory HIT. Serum of patients with HIT that was mixed with IVIg showed decreased platelet activation by HIT antibodies compared with the control. Padmanabhan et al further demonstrated that the immunoglobulin G Fc domain of infused antibodies competed with HIT antibodies for the platelet receptor FcγRIIa, in turn inhibiting platelet activation.^14^ This mechanism of action prevents further platelet consumption, leading to resolution of thrombocytopenia and prevention of thrombosis.^6^ Additional suspected mechanisms of action include inhibition of antibody production, and direct inactivation of HIT antibodies via anti-idiotypic antibodies.^23^ IVIg carries the US Food and Drug Administration black box warning for thrombosis in hypercoagulable states such as cancer^23^; however, there were no reports of new or worsening thrombosis after IVIg in any of the cases of refractory HIT.^7,23,24^

In contrast to most case reports, our patient showed no response to initial 2 doses of IVIg. However, Doucette et al reported a similar case in which initial recovery after 2 doses of IVIg was followed by platelet count decline. Repeat ELISA and serotonin release assay confirmed the persistence of antibodies, and the patient responded to repeat IVIg therapy.^7^ Although we did not repeat HIT testing, this is consistent with our experience.

### Corticosteroids

Corticosteroids are not currently a recognized treatment modality for refractory HIT. Methylprednisolone was administered to this patient to hypothetically suppress the autoimmune component by inhibiting ongoing antibody synthesis. Literature search found 2 cases that utilized corticosteroids, albeit with uncertain benefit as it was administered alongside IVIg.^7^

## Conclusion

This case serves to suggest a potential benefit of changing anticoagulation in cases of autoimmune HIT when initially unresponsive to IVIg. Nevertheless, a retrial of IVIg is also warranted due to possible antibody persistence. Given that anticoagulation poses a risk when treating HIT with cerebral venous sinus thrombosis or other high bleeding risk conditions, it may behoove us to consider IVIg earlier in the course of such patients. Further research should be directed to establish the efficacy of IVIg in refractory HIT and to determine its utility in early treatment of HIT in high bleeding risk cases. Additionally, our case serves as a reminder to use HIT risk stratification to guide platelet monitoring and to consider the use of DOACs as an alternative to heparin products.
